# Percutaneous Coronary Interventions in Nonagenarians: Single-Centre Insights

**DOI:** 10.3390/jcm14207371

**Published:** 2025-10-18

**Authors:** Gwidon Polak

**Affiliations:** Faculty of Medicine, Bydgoszcz University of Science and Technology, Aleje Prof. S. Kaliskiego 7, 85-796 Bydgoszcz, Poland; gwidon.polak@pbs.edu.pl

**Keywords:** coronary angiography, percutaneous coronary intervention, nonagenarians

## Abstract

**Background/Objectives**: Despite the common use of invasive diagnostics and treatment of coronary artery disease (CAD), there are still doubts concerning the disease management method of choice in the population of very old patients. Our goal was to assess the patient profile, feasibility of coronary angiography (CAG), effectiveness (successful relieving of the coronary artery’s narrowing or occlusion) of percutaneous coronary intervention (PCI) and safety (mortality and other complications) of both procedures in nonagenarians. **Methods**: The database of the Dr. E. Warmiński Clinical Hospital of the Bydgoszcz University of Technology was searched for patients aged 90 years and older who underwent CAG and PCI between 2013 and 2023. We retrospectively analysed the case reports of these patients, including reason for hospital admission, course of hospitalisation, procedure data, and complications. **Results**: A total of 150 nonagenarians meeting the criteria were found, with a mean age of 92 years and 63% being female. A total of 110 patients (73%) were admitted on the basis of acute coronary syndrome (ACS). Upon CAG, 108 patients had obstructive coronary artery disease confirmed, 90% of whom had multivessel disease. In 96 out of 108 of these patients (that is, 89%), PCI was performed successfully in 89 (93%) procedures. Transradial access was used in 112 patients (75%). According to the diagnosis, PCI was performed in all cases (100%) of STEMI patients, in 80% cases of non-ST elevation acute coronary syndrome (NSTE-ACS) patients, and in 27% cases of stable CAD patients. Median time of hospitalisation was 6.5 days (IQR 4–10). In the course of hospitalisation, mortality was 8.7% (13 out of 150), although two cases were non-cardiological in nature. In the PCI group, mortality was 11.5% (11 out of 96); all 11 were treated due to ACS (no deaths in patients with stable ACS). In the STEMI subgroup, mortality was much higher at 33% (4 out of 12, with all 4 admitted with cardiogenic shock). Accordingly, in the NSTEMI group, mortality was 8.97%. Other complications in the PCI group were perforation of coronary artery in 1 case, access site complications in the case of transfemoral access in 10 patients, bleeding requiring transfusion in 2 patients, and contrast-induced nephropathy (CIN) in 4 patients. **Conclusions**: This analysis demonstrates that the CAG and PCI procedures are feasible and effective in nonagenarians, and the risk of complications is not as great as it was heretofore believed.

## 1. Introduction

The world population is experiencing ageing on an unprecedented scale, which is a particular concern in Western countries. What deserves attention is the worldwide ageing of the older population itself; in Europe alone, the number of people aged 85 years or more between 2019 and 2050 is projected to more than double, up 113.9% [1]. This population is becoming a growing group of patients in everyday practice, posing unique challenges to modern medicine, among them multimorbidity, frailty syndrome, varying degrees of cognitive impairment, and increased risk of procedure-related complications [2].

Cardiovascular diseases (CVDs), especially coronary artery disease (CAD), remain the leading cause of morbidity and mortality in the elderly population [2]. In Poland, CVDs accounted for 44.87% of morbidity and mortality in men and 51.84% in women among people aged 75 or older in 2019 [3]. For years now, management of CAD has been based on coronary angiography (CAG) and, when indicated, subsequent percutaneous coronary interventions (PCIs); however, elderly patients are all too often disqualified from interventional treatment on the basis of comorbidities, cognitive impairment, frailty syndrome, limited life expectancy, and others. For the same reasons, patients from this group are underrepresented in randomised trials [2,4,5], leading to further lack of data concerning invasive management of CAD in the elderly. Another difficulty is the increased risk of complications, further affecting the choice of treatment method and clinical outcome in this age group.

As quality of life in the elderly steadily increases and with advances in the field of invasive management of CAD promising safer and more efficient treatment, a reassessment of the heretofore approach seems necessary. However, there is a lack of randomised trials concerning the safety and efficacy of invasive management of CAD in the elderly, especially nonagenarians (patients aged ≥ 90 years). While there are observational studies specifically on the subject (both single- and multi-centre ones), no similar study based on the Polish population has been published thus far [2,6,7,8].

This retrospective study aimed to analyse the clinical presentation, risk factors, comorbidities, complications, and mortality in patients 90 years or older who underwent CAG and PCI at our centre over a period of 11 years. We also aimed to analyse procedural data of CAG and PCI, their feasibility, effectiveness (success rate), and related complications.

## 2. Patients and Methods

The database of the Dr. E. Warmiński Clinical Hospital of the Bydgoszcz University of Technology was retrospectively reviewed for patients who underwent coronary angiography (CAG) and percutaneous coronary intervention (PCI) over a period of 11 years (between 1 January 2013 and 31 December 2023). From the entire group of 14,176 patients, 150 nonagenarians meeting the aforementioned criteria were identified (0.1%). The baseline characteristics including risk factors and comorbidities were analysed, including age, gender, arterial hypertension (defined as SBP > 140 or DBP > 90 mmHg or taking antihypertensive medications), diabetes mellitus (HbA1c > 6.5% or taking antidiabetic medications), hyperlipidaemia (LDL-cholesterol > 70 mg/dL or triglycerides >150 mg or taking lipid-lowering medications), cigarette smoking (patient statement), obesity (BMI ≥ 30), chronic renal failure (GFR < 60 mL/min), anaemia (Hb < 12 g/dL), peripheral artery disease, and chronic obstructive pulmonary disease (COPD).

Furthermore, the following relevant cardiovascular conditions were assessed prior to the procedure: previous myocardial infarction, PCI, and coronary artery bypass grafting (CABG); atrial fibrillation (AF) and presence of cardiac implantable electronic device (CIED); and previous stroke and transient ischemic attack (TIA). Clinical presentation and definitive diagnosis were similarly reviewed. Definitive diagnosis was established based on anamnesis, clinical examination, ECG results, and serial measurements of cardiac troponin level (hsTn). Myocardial infarction was diagnosed according to the 4th Universal Definition of Myocardial Infarction, which was then further narrowed down to STEMI or non-STEMI based on ECG results. Patients with symptoms of acute myocardial ischaemia and ischaemic changes in ECG but normal hsTn values were accordingly diagnosed with unstable angina.

We analysed peri- and procedural data and complications (if any), as well as the outcome of PCI and the whole period of hospitalisation recorded in the database.

Descriptive data is presented as means with standard deviation (SD) or medians with interquartile range (IQR), while categorical variables are reported as percentages. The normality of distribution variables was assessed using the Shapiro–Wilk test.

This study was approved by the Bioethical Commission at the Faculty of Medicine, Bydgoszcz University of Science and Technology (No. 4/2025), and conducted in accordance with the principles of the Declaration of Helsinki.

## 3. Results

### 3.1. Study Population

The baseline characteristics, comorbidities, and laboratory findings of the study population were analysed. The mean patient age was 92 years; 95 (63%) of the patients were female. The demographic and clinical characteristics are presented in Table 1.

In the group of 150 patients, 143 had echocardiography performed. Left ventricular ejection fraction (LVEF) below 50% was found in 58 patients (40%). Severe aortic stenosis was present in nine patients, severe mitral regurgitation in eight patients, and severe tricuspid regurgitation in 12 patients (accordingly, 6, 5, and 8%).

Next, data on diagnosis and subsequent disease management of the study population was analysed. The indications for CAG are shown in Table 2. The most frequent initial diagnosis was acute coronary syndrome (ACS): 110 in the whole group (73%). The definitive diagnosis was STEMI in 12 (8%), NSTEMI in 80 (53%), and unstable angina in 18 (12%) patients, resulting in a total of 98 patients (65%) with non-ST-segment elevation acute coronary syndrome (NSTE-ACS). A total of 22 (15%) patients were diagnosed with stable coronary artery disease (chronic coronary syndrome), six (4%) with heart failure, four (2.7%) with arrhythmias, and four (2.7%) with valvular heart disease. Other primary diagnoses concerned the last four (2.7%) patients.

### 3.2. Procedural Data

Procedural and angiographic data is presented in Table 3. All 150 patients underwent CAG. We used transradial access (TRA) in 112 patients (75%) and transulnar access in one patient. Transfemoral access was employed in 37 patients (24.6%), though in 6 of those cases it was after failure of primary TRA. The arterial sheath size used was 6-Fr.

A total of 108 patients (72%) had obstructive coronary artery disease confirmed upon CAG. Intermediate stenosis (of up to 50%) was found in 13 patients (9%). In the remaining 29 patients (19%), no stenosis was found (including 9 patients who had undergone PCI in the past, in which no in-stent restenosis was found).

In the group with obstructive coronary artery disease present, the majority of patients displayed multivessel disease—97 out of 108 (90%). In the other 11 patients (10%), single-vessel disease was found. In nine patients (8%), a significant stenosis of the left main trunk was discovered. Percutaneous coronary intervention (PCI) was performed in 64% patients (96 out of 150) who underwent CAG, and in 89% (96 out of 108) patients with confirmed CAD (the other 11% were treated conservatively). In cases of multivessel disease, only the culprit lesion (the lesion responsible for the symptoms) was treated. This data is presented in Table 4.

**Figure 1:**
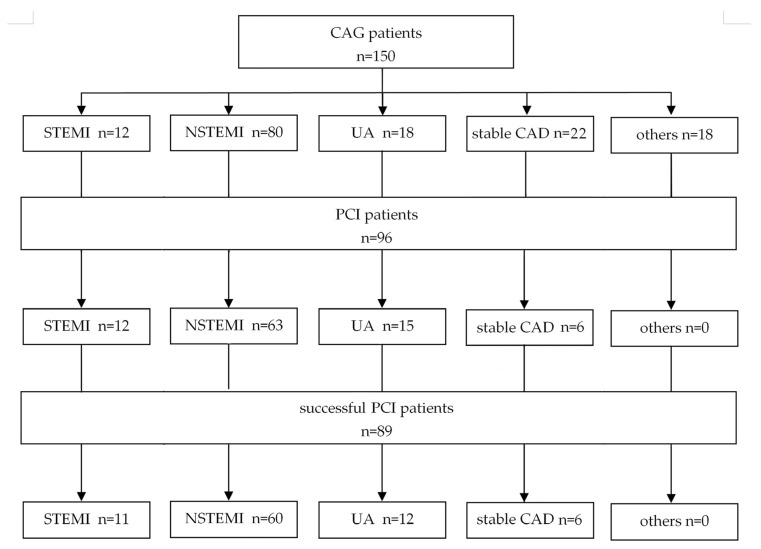
Study flow chart. CAD = coronary artery disease; CAG = coronary angiography; NSTEMI = non-ST-segment elevation myocardial infarction; PCI = percutaneous coronary intervention; STEMI = ST-segment elevation myocardial infarction; and UA = unstable angina.

Taking primary diagnosis as differentiation criteria, out of the group of 110 patients diagnosed with ACS, 90 patients (82%) had PCI performed; in the group of 12 patients diagnosed with STEMI, PCI was performed in all cases (100%); and in the group of 98 patients diagnosed with NSTE-ACS, PCI was performed in 80% cases (78 out of the remaining 98 patients). A total of six PCI procedures were performed in patients diagnosed with stable CAD (27% out of 22 patients with that diagnosis). This data is presented in detail in Figure 1.

Among 96 performed PCI procedures, 89 (93%) were successful: in 85 cases, at least one stent was implanted (in 32 cases, two or more stents were implanted), in 3 cases of in-stent restenosis (ISR), plain old balloon angioplasty (POBA) or angioplasty with drug-eluting balloon (DEB) was performed. PCI treatment with stents was successful in nine patients with significant stenosis of the left main trunk. In one case, manual thrombectomy only was performed due to the embolism occurring from atrial fibrillation. Due to severe calcification, 12 persons had rotablation performed. Intracoronary ultrasound was used in five patients. A total of seven PCI procedures were unsuccessful. None of the patients had to be referred to coronary artery bypass grafting (CABG). In Table 5, we present the data concerning PCI procedures.

### 3.3. Outcome and Complications

The median time of hospitalisation was 6.5 days (IQR 4–10). We analysed all complications that appeared over the course of hospitalisation. In the whole group of 150 patients, 13 (8.7%) died during hospitalisation. Two patients died due to non-cardiological causes, having presented no significant coronary artery lesions in CAG and no complications related to the procedure itself. The remaining 11 patients were treated with PCI due to acute coronary syndrome: 4 had STEMI and 7 had NSTEMI. No patient with stable CAD died during hospitalisation, even those undergoing PCI (Table 6).

Concerning the whole group of 96 patients who underwent PCI, the percentage of mortality was 11.5% (11 out of 96); in the subgroup of patients treated with PCI due to ACS (90 patients), the mortality percentage was 12.2% (11 out of 90 patients died); in the STEMI subgroup, 33.3% (4 out of 12 patients died); due to NSTE-ACS, 8.97% (7 out of 78 patients with NSTE-ACS died, all 7 of them diagnosed with NSTEMI); and in the stable CAD subgroup, no patients died.

The primary cause of death in the STEMI subgroup (four patients) was cardiogenic shock with pre-admission onset (three out of four patients died during PCI or within 24 h of it). In the NSTEMI subgroup (seven patients), cardiogenic shock was the cause of death in two patients, asystolic cardiac arrest (within a few days after PCI) in three patients, and respiratory failure due to severe pneumonia in two patients. This data is presented in Table 7.

We observed one case of perforation of the coronary artery during PCI, which was successfully treated via stent graft implantation. Access site complications occurred in 10 patients with transfemoral access, all after PCI: 8 had a haematoma and 2 had a pseudoaneurysm, and 2 needed blood transfusion. In the PCI group of 96 patients, 4 developed contrast-induced nephropathy (CIN), which was treated conservatively, 12 were newly diagnosed with atrial fibrillation, and 3 were diagnosed with atrioventricular block requiring pacemaker implantation. Complications are presented in Table 8.

In 54 patients who underwent CAG, but not PCI, there were no complications except only one CIN, which was treated conservatively. No case of stroke or myocardial infarction related to CAG or PCI was observed.

In the group of all studied patients, *n* = 150, pneumonia was a relatively common complication—we found it in 12 patients (8%). A total of nine patients were referred for transcatheter aortic valve implantation (TAVI).

## 4. Discussion

Our study found that in our centre’s catheterisation laboratory, nonagenarians have generally been admitted due to ACS, especially NSTEMI. All of these patients could feasibly have had CAG performed. The most common finding was multivessel disease, but the culprit lesion could have been established and successfully treated via PCI with stent implantation, with a low complication rate.

Populations worldwide are experiencing heretofore unprecedented ageing, which means that the number of very elderly patients undergoing CAG and PCI is steadily increasing. However, very elderly patients, particularly nonagenarians, remain a largely unstudied population, leaving uncertainty regarding the treatment of choice and its safety. It should be noted that many elderly patients maintain a good quality of life and thus pursue interventions to preserve their health, highlighting both the clinical and societal importance of clarifying the role of PCI in nonagenarians.

Our single-centre study encompassed 150 nonagenarians, who accounted for 0.1% of all patients undergoing CAG and PCI over the course of 11 years. In the studies of other centres, these figures are likewise low, albeit comparatively higher—from 0.25 to 0.9% [6,7,8]. Women predominated in our study group, which corresponds with data presented by other authors [9,10,11]. It is noteworthy that, with the exception of arterial hypertension, cardiovascular risk factors were rare in our population, especially obesity and smoking; this has also been observed in other studies [6,12]. It is the absence of most risk factors that may enable such a long life in the study group (due to natural selection). On the other hand, other comorbidities were much more common, especially renal failure and anaemia. Our study population frequently suffered from atrial fibrillation and reduced left ventricular ejection fraction (LVEF < 50%). The findings described above are well-documented in the elderly [2,9,11].

The median time of hospitalisation was 6.5 days (IQR 4–10), which is longer than that reported by other authors [2,10]. This may be related to the multimorbidity of our studied group, as described above.

The most common indication for CAG in the analysed group was ACS: 110 out of 150 patients (73%), with NSTEMI being the most common (over 50%). Of this number, as many as 90 (82%) underwent PCI, which was effective in over 90% of cases, with a low complication rate and in-hospital mortality of about 12%. In 90% of patients, multivessel disease was documented in CAG. These findings are similar to those in previously published studies, although the proportion of STEMI in ACS was usually higher than in our group [7,8,11,12]. Multivessel disease also predominates in these studies, although the number of left main artery disease cases varied greatly.

The PCI procedures we performed on our patients mainly involved stent implantation, with one stent being sufficient in over half of the cases. Despite the common presence of severe calcification in the elderly population, we used rotablation in only 13% of PCI cases, as—in previous years—it was not available; the same applied to IVUS. However, since then, usage of both IVUS and rotablation has become a standard procedure in our centre.

Invasive treatment of CAD in elderly patients has long been controversial and remains difficult to evaluate [5]. The risk of complications from CAG and PCI procedures, both ‘mechanical’ and those related to the administration of contrast (CIN) and anticoagulants (bleeding), is indeed higher in the elderly than in younger patients [5,13]. In our material, there were no cases of stroke or TIA. Five cases of CIN (one after CAG and four after PCI) were recorded (though no dialysis was necessary), which represents 3% of the 150 CAGs performed and is lower than the data reported in older studies [7]. During hospitalisation, we observed frequent new-onset cases of atrial fibrillation, specifically 12 patients, which is 12.5% in the PCI group (*n* = 96) and 8% in the entire studied group (*n* = 150). Moreover, pneumonia was observed in 12 patients (8% in the entire studied group, *n* = 150). Both of these findings are also described by other authors [7].

The transradial approach was successful in 75% of patients, which largely accounted for the reduction in bleeding complications in the entire group. Access site complications concerned only femoral access and PCI procedures; however, considering that this applies to a group of 38 patients (since the transradial approach was used in 112 patients), complications were not rare cases in this subgroup (a total of 10 patients with various forms of local complications). This may indicate the susceptibility of nonagenarians to complications associated with femoral artery puncture. Although paradoxically, since interventional cardiologists with high proficiency with the radial route tend to be associated with worse outcomes of PCI via the femoral artery [14], it is this phenomenon that may be responsible for the increased number of complications in the femoral access subgroup. Bleeding requiring transfusion occurred in 2% of patients who underwent PCI, which is less than described in the literature [7,11]. Other complications typical of PCI procedures were sporadic in our material (coronary artery perforation was found in one patient).

In-hospital mortality in the entire group that underwent CAG was 9%, in the subgroup that underwent PCI it was 11.5%, and in patients with an ACS diagnosis it was 12.2%. In similar single-centre studies, in-hospital mortality varied between 10 and 15.7% [7,11,12]. In a very large study of approximately 70,000 nonagenarians undergoing PCI in the United States, the indication for PCI was STEMI in about 28% of patients, NSTEMI in 50%, and stable CAD in 22%. In-hospital mortality was 16.4% in STEMI, 4.2% in NSTEMI, and 1.8% in stable CAD [8]. In our studied group, we performed 96 PCI procedures: 90 in patients with ACS (the majority of them with NSTEMI) and 6 in patients with stable CAD. Patients with STEMI constituted a smaller group than in other studies (12 patients, i.e., only 8% of all 150 patients undergoing coronary angiography). These were the most severely ill patients; four of them, i.e., 33%, died over the course of hospitalisation, with three patients dying on the day of hospital admission from cardiogenic shock. In-hospital mortality in NSTE-ACS was 8.97% (7 patients with NSTEMI out of 78 with NSTE-ACS died). Among those with stable CAD, no patients died. In a single-centre study similar to ours, conducted by Finnish authors, the indication for PCI was STEMI in 33%, other acute coronary syndrome in about 50% of patients, and stable CAD in 20%. In that study, mortality was mainly related to STEMI: in-hospital mortality in these patients was 22.9%, 5.9% in NSTE-ACS, and 0% in stable CAD [11]. In another recent study, mortality in STEMI treated with PCI in nonagenarians was about 27% [10].

While it is true that older patients with acute myocardial infarction have a worse prognosis than younger patients, recent studies have confirmed that primary invasive PCI treatment improves it [9,15,16]. In a study published in 2022, which analysed more than 58,000 patients with STEMI treated between 2010 and 2018 in the US, primary PCI mortality during hospitalisation was 15.8% versus 32.2% in the group treated without PCI [17]. Another analysis published in 2025, covering over 300,000 patients in the US diagnosed with acute myocardial infarction, confirms lower mortality in patients treated with PCI compared with those treated with optimised medical therapy (OMT): 9.9% versus 16.2%. It should be emphasised that in the aforementioned study, the vast majority of patients in the analysis were treated with OMT [18]. A Polish report published in 2023 on the treatment of acute myocardial infarction in nonagenarians in the period from 2014 to 2020 showed that only 47% of these patients underwent invasive management (at least CAG) and only 35% underwent PCI; in-hospital mortality was 27.8% [19].

In 2025, a meta-analysis enrolling over 100,000 patients was published, comparing primary PCI with conservative treatment in older people (≥80 years) with STEMI [20]. A total of 98% of patients included in the study were nonagenarians. The overall survival rate was 76.5% in the PCI group and 67.2% in the conservatively treated group, which was a statistically significant outcome (*p* < 0.01). In-hospital mortality was 15% in the PCI group and 28.9% in the conservatively treated group (*p* < 0.01), while 1-year mortality was 29.1% in the PCI group and 54.4% in the conservatively treated group (*p* < 0.01). There was no difference in the prevalence of major bleeding between the two groups. It is worth noting that in the study, only about 30% of patients diagnosed with STEMI were treated invasively. These findings point to a potential underuse of PCI in older patients with STEMI. The authors concluded that real-world data from registries and observational studies indicate that percutaneous coronary intervention should not be automatically withheld from older patients.

Qualification for CAG and PCI appears to be significantly more difficult in patients with stable CAD. In our material, these patients accounted for only 15% of those undergoing CAG (22 patients), of whom only 6 (27%) underwent PCI. This indicates the need for caution in assessing the risk–benefit ratio and subsequent qualification for invasive treatment. Nevertheless, there is a group of nonagenarians who may benefit from PCI in stable CAD, especially in terms of improving their quality of life [21]. The procedures performed in this group of patients in our centre were effective and without complications.

As our study was single-centre and retrospective, we acknowledge several limitations. One limitation is the potential bias in patient selection; because our study concerns only patients treated interventionally, we do not know which part of the entire nonagenarian population admitted to our centre’s cardiology department they represent. The lack of follow-up is another significant limitation of our study, especially concerning a 30-day post-discharge mortality assessment.

## 5. Conclusions

The data analysed and described above supports the advisability of invasive treatment of acute coronary syndromes in nonagenarians and indicate the need for a change in existing clinical practice concerning the disease management method of choice in that population. We found that CAG can be feasibly performed, and the culprit lesion can be identified (despite the predominance of multivessel disease) and treated successfully in almost the entire studied population. Considering the patient profile of nonagenarians, complications associated with interventional procedures and in-hospital mortality seem to be within an acceptable range. Our data also indicates that selected patients with stable coronary artery disease can be feasibly and safely treated with PCI. In the face of a steadily ageing population and increasing quality of life in the elderly, the importance of the discussed subject will only grow. As such, further research in the field of diagnostics and treatment of coronary artery disease in the elderly seems necessary.

## Figures and Tables

**Table 1:** Demographics and clinical characteristics.

All Patients *n* = 150	
Characteristics	N (%)
Age—years (mean)	92 ± 1.79
Female	95 (63)
Hypertension	121 (81)
Diabetes mellitus	58 (39)
Hyperlipidaemia	77 (51)
Cigarette smoking	1 (0.7)
Obesity (BMI ≥ 30)	24 (16)
Renal failure	85 (57)
Anaemia	59 (39)
COPD	9 (6)
Peripheral artery disease	14 (9)
Previous stroke/TIA	19 (13)
Previous MI	42 (28)
Previous PCI	50 (33)
Previous CABG	4 (2.5)
Atrial fibrillation	55 (37)
CIED (PM/ICD)	19 (18/1) (13)
LVEF below 50%	58 (40)
Severe AS	9 (6)
Severe MR	8 (5)
Severe TR	12 (8)

The data is presented as number (%) for categorical values and mean ± standard deviation for continuous variables. AS = aortic stenosis; BMI = body mass index; CABG = coronary artery bypass grafting; CIED = cardiac implantable electronic device; ICD = implantable cardioverter defibrillator; COPD = chronic obstructive pulmonary disease; LVEF = left ventricular ejection fraction; MI = myocardial infarction; MR = mitral regurgitation; PCI = percutaneous coronary intervention; PM = pacemaker; TIA = transient ischemic attack; and TR = tricuspid regurgitation.

**Table 2:** Indications for procedure.

All Patients *n* = 150	
Indications	N (%)
Stable CAD	22 (15)
STEMI	12 (8)
NSTEMI	80 (53)
Unstable angina	18 (12)
Heart failure	6 (4)
Arrhythmias	4 (2.7)
Valvular heart disease	4 (2.7)
Other	4 (2.7)

The data is presented as number (%). CAD = coronary artery disease; NSTEMI = non-ST-segment elevation myocardial infarction; and STEMI = ST-segment elevation myocardial infarction.

**Table 3:** Procedural and angiographic data in the entire group.

All Patients *n* = 150	
Transradial access	112 (75)
Transulnar access	1 (0.7)
Obstructive coronary disease	108 (72)
No significant lesions (up to 50%)	13 (9)
No changes, without ISR	29 (19)
PCI after CAG	96 (64)

The data is presented as number (%). ISR = in-stent restenosis; PCI = percutaneous coronary intervention; and CAG = coronary angiography.

**Table 4:** Angiographic data in patients with obstructive coronary disease.

The Group with Obstructive Coronary Disease *n* = 108	
Angiographic Data	N (%)
One-vessel disease	11 (10)
Multivessel disease	97 (90)
Left main trunk disease	9 (8)
PCI after CAG	96 (89)
Successful PCI	89 (93)

The data is presented as number (%). CAG = coronary angiography; PCI = percutaneous coronary intervention.

**Table 5:** PCI procedural data.

The PCI Group *n* = 96	
PCI Procedural Data	N (%)
STEMI	12 (12.5)
NSTE-ACS	78 (81)
Stable CAD	6 (6.25)
Successful PCI	89 (93)
PCI with stent implantation	85 (89)
- One stent	53 (55)
- Two or more stents	32 (33)
PCI—POBA (in case of ISR)	2 (2)
PCI with DEB (in case of ISR)	1 (1)
Thrombectomy only	1 (1)
Rotablation	12 (13)
Use of IVUS	5 (5)

The data is presented as number (%). CAD = coronary artery disease; CAG = coronary angiography; DEB = drug-eluting balloon; ISR = in-stent restenosis; IVUS = intravascular ultrasound; NSTE-ACS = non-ST-segment elevation acute coronary syndrome; PCI = percutaneous coronary intervention; POBA = plain old balloon angioplasty; and STEMI = ST-segment elevation myocardial infarction.

**Table 6:** Mortality (all-cause) as referred to clinical presentation.

In-Hospital Death *n* = 13	
Non-cardiologic group	2
Stable CAD group	0
PCI group	11 out of 96 (11.5%)
- PCI in ACS group	11 out of 90 (12.2%)
- PCI in the STEMI group	4 out of 12 (33.3%)
- PC in the NSTEMI group	7 out of 78 (8.97%)

The data is presented as number (%). ACS = acute coronary syndrome; CAD = coronary artery disease; NSTEMI = non-ST-segment elevation myocardial infarction; PCI = percutaneous coronary intervention; and STEMI = ST-segment elevation myocardial infarction.

**Table 7:** Cause of death in patients undergoing PCI.

The PCI Group *n* = 96	
In-hospital mortality	11 (11.5)
Death within the first 24 h after PCI	3 (3)
Cardiogenic shock	6 (6)
Asystolic cardiac arrest	3 (3)
Respiratory failure	2 (2)

The data is presented as number (%). PCI = percutaneous coronary intervention.

**Table 8:** Complications of PCI and during hospitalisation.

The PCI Group *n* = 96	
In-hospital mortality	11 (11.5)
Perforation of the coronary artery	1 (1)
Access site complication:	
- Haematoma	8 (8)
- Pseudoaneurysm	2 (2)
Bleeding requiring transfusion	2 (2)
CIN	4 (4)
Atrial fibrillation	12 (12.5)
Atrioventricular block	3 (3)

The data is presented as number (%). CIN = contrast-induced nephropathy; PCI = percutaneous coronary intervention.

## Data Availability

The data underlying this article will be shared upon reasonable request to the corresponding author.
